# Odontological Society of Pennsylvania

**Published:** 1892-12

**Authors:** 


### ODONTOLOGICAL SOCIETY OF PENNSYLVANIA.

The regular meeting was held October 8, 1892, at the rooms of the Society, 1228 Walnut Street, Philadelphia, President Louis Jack, presiding. After the transaction of the usual routine business, a paper by Dr. W. G. A. Bonwill, of Philadelphia, was read on the following subject: "Mechanics in Dentistry.—Is Dentistry a Science or a Profession?"

(For Dr. Bonwill's paper, see page 860.)

The President called upon Dr. Truman to open the discussion.

*Dr. Truman.*—There is not much to discuss in the paper, as I view it. I think we can agree with Dr. Bonwill that we are all mechanics in one sense, but because we are so it does not follow that we are not professional men. We can combine the two. I do not regard it possible to practise dentistry without being scientific, —without being something more than a mere worker in tools. A single tooth in the mouth cannot be treated without understanding the very science derided here to-night, and the moment the operator steps aside from that, just at that moment he is a failure in dentistry. To me the argument made is simply a plea for a special line of work.

We belong, I believe, to a growing profession, and I hope to see it develop into one of the great professions of the world. I believe we have in this work very much that is truly scientific,—more involved in it that is good for humanity than almost any other. Medicine is not science; never has claimed to be. There are specialties in medicine that are truly so, and ours is one of them. I contend that we, as dentists, have just as much right to the title doctor as any man in medicine. I am unwilling to lower my flag to any medical man, no matter how skilful he may be.

To practise our profession, we must be mechanics, and I am proud of it. I can never look at one of the great buildings in this city without veneration for the man that has reared it. Standing under the dome of St. Paul's, London, I could feel the power of the individual that laid one stone upon another, but because I thus feel is it required of me that I should abandon the position gained by greater effort? I object to it.

It is no wonder Dr. Bonwill did not value his degree; he never earned it. If he had gone through the schools day after day, sitting on the hard benches, and had studied along with others, he would have appreciated the feeling all have as students and as co-laborers. He came into the profession through another door, and there I leave him.

*Dr. Leffman.*—I do not know that I can add anything to the discussion. I feel very much as Dr. Truman does, that there are two sides to the profession, which certainly is regarded generally as a mechanical one.

I do not agree with the inferential condemnation of purely scientific students, which I gather from the paper. I believe the advances made in bacteriology have been of great value, although it may not at the present moment appear quite so clearly to everybody, particularly with practical men, who have learned to deal with problems in the operating-room. I think it is of great value in modifying the methods of dentists.

When the bacteriological discoveries were announced years ago they were smiled at by many, but are now acknowledged to be of value. Even the late development and discovery of lymph, as it was called, after passing through a period of contempt and condemnation, is now coming to be recognized as a most valuable aid in the treatment of a most formidable affection. The work of research in our practice is not as brilliant in its results as some other departments, by reason of the narrowness of the field, but it will certainly have its effect. I have myself, as a teacher in a dental college, been interested for years in everything connected with its advancement, and I therefore cannot deny that a large part of dentistry is mechanical, as it is also a part of surgery. The most brilliant work of surgery is of that character, but we must not overlook the purely professional feature.

I am inclined, therefore, simply to second Dr. Truman's remarks that the dentist is entitled to the degree of doctor, and by encouraging the development of the dental student, and giving him something of a professional standard to attain, we develop his usefulness and make him far more serviceable in the practice of his profession.

We may leave the purely mechanical features to their natural development. The human mind, especially the American, runs largely in that direction. I do not think we will have any want of improvement in this by reason of any development of theoretical or professional features.

*Dr. Jeffries.*—It seems so me that the key-note has hardly been struck, or, at least, I haven't heard anybody take the position I think the question should occupy. Dentistry, as practised at the present day, is something that stands entirely by itself. It is professional; it is mechanical; it is artistic. You cannot practise successfully without bringing all into use. An exponent of the art in the highest degree must have attributes which every man cannot possess. He must be thoroughly learned in manipulative skill, which he can only acquire by proper teaching in his college and in his office; if he has cases to regulate, it will require the highest degree of mechanical skill, and it does seem to me that to call it either the one or the other is a mistake, as it will need both to make it a success.

*Dr. Darby.*—I think Dr. Bonwill was pleading to-night for only one side of the question. I do not think that away down in his heart he repudiates the theoretical part of dentistry. He was talking on only one side of it. We all know that it is out of the abundance of the heart the mouth speaketh. He is a mechanical inventive genius, and it is natural he should dwell in that direction, but I do not think he repudiates the idea of the value of therapeutics or bacteriology in the management of dental lesions or in the treatment of teeth. I should be very sorry to think he was so biased in one particular direction, or that he had so little sense, if I may say so, as to wish to bring that paper as an epitome of the intelligence of the profession. It certainly is not.

*Dr. Guilford.*—I doubt if Dr. Bonwill expressed himself as clearly as he might have done. When making his special plea for mechanics he seemed to think it was something separate from science, which is a great mistake.

Dr. Bonwill is well acquainted with mechanics, especially dental mechanics, but he never did a single thing, he never invented any instrument, without there being science at the bottom of it. I don't see how the two can be divorced. They belong together. There is not a motion in mechanics but has associated with it the elements of science; so that when we consider the dental profession, I think the majority of us would not be willing to agree to what Dr. Leffman has said, that it is a specialty of medicine, and we are rather inclined to call it a branch of the healing art.

There are certain things connected with the practice of dentistry which make it different from any other profession or calling. We treat cases as medical men to a limited degree. We are mechanics in filling teeth or making a plate. If we do our work in an artistic and skilful manner, if we perform all the different things we are called upon to do, I cannot see why we are not scientific and artistic, because both of them are really connected in mechanics; so that I should say that the professional dentist includes not only mechanics and art, but science also. Some of the work is very difficult,—more so than much of that done by some of our sister professions. The surgeon's amputation of a limb or removing a bone requires a certain amount of manipulative skill and scientific knowledge, or it could not be done successfully, and yet we have surgical work that is more difficult than the removal of a bone, setting a limb, or amputation.

Take, for instance, the branch of orthodontia where the positions of the teeth require changing. It seems to me that this includes all the different elements we have been speaking of to-night. Appliances must be made to move the teeth. The lines of force must be exactly known, and also the power of resistance and the correlation of forces. Is not all that scientific? Does not that introduce mathematics in a very large degree. And when a tooth is to be moved, it cannot be performed as a surgeon would, by a simple surgical operation. It must be accomplished in a very slow manner indeed. Forces must be applied, and certain changes must be depended upon which will enable the tooth to move. It has to be performed with skill, care, and caution. After that we are obliged to retain the teeth in place. So I believe there is more difficult surgery involved in the correction of a case of irregularity than in the amputation of a limb.

Dentistry occupies a very large field and brings in different sciences and arts, and if this be true I think we occupy a position by ourselves. Anything that is made up of science and art is a profession, and it is nothing else.

I agree entirely with Dr. Bonwill's plea for mechanics, but I think he has not made it broad enough, because it includes a great deal more than that. He spoke, too, of the fact that students were not willing to take up the mechanical part. That is certainly true to a large extent, and I think that difficulty has been recognized by the teachers of to-day, and arrangements have been made by which they shall be taught this branch more fully than they have been before. I know it is so in many colleges; it may be so in all. As soon as a want or a deficiency of that kind is felt, arrangements are immediately made to remedy it, to turn out, if possible, better and more scientific and skilful dentists than have ever been graduated in the past.

*Dr. Bonwill.*—In answer to criticisms and remarks made by the members, I have to correct the very false idea raised by most of them that I could not see that dentistry could be more than mechanical. While I can count upon my fingers the number of my medicinal remedies, I am not lost to the fact that a man should be well educated and have passed through a medical training, not alone for the remedies used, but for the higher culture it gives to one, and for the better understanding the laws of inflammation and the aseptic treatment. The remedies are very few that are actually needed for any and all the diseases of the teeth and adjacent parts, yet they should be intelligently understood to be made effective.

What I mean is, that above all else we must admit that mechanics in dentistry is the basis of nearly all our needs and successes, and that when we would sink this principle out of sight, fearing to face the world with such a name, we are doing that which is unworthy of our calling.

Much stress has been laid on the treatment of caries by asepsis, as if it were the *sine qua non.* Professor Tait, of Scotland, demonstrated that he had greater success with cleanliness than by the medicines for aseptic treatment, and Professor Lister had to acknowledge at Berlin, in 1890, to the mistake he had made in attributing so much to carbolic acid and antiseptics.

I would not for a moment have you simply be a mechanic in dentistry. In dealing with the human economy we must have as much information from study as will enable us to put our mechanical skill to the proper use; law cannot be successful unless a man has ingenuity or power of construction.

Invention gives in this line of work a power not attained outside. We cannot deny that should we lose our hands we would be helpless. Manipulation is mechanical dexterity, and let us call it by the right name,—not *prosthesis.*

